# Biodegradable Microparticles for Regenerative Medicine: A State of the Art and Trends to Clinical Application

**DOI:** 10.3390/polym14071314

**Published:** 2022-03-24

**Authors:** Anastasia A. Sherstneva, Tatiana S. Demina, Ana P. F. Monteiro, Tatiana A. Akopova, Christian Grandfils, Ange B. Ilangala

**Affiliations:** 1 Enikolopov Institute of Synthetic Polymeric Materials, Russian Academy of Sciences, 70 Profsouznaya Str., 117393 Moscow, Russia; sherstneva2912@mail.ru (A.A.S.); akopova@ispm.ru (T.A.A.); 2 Institute for Regenerative Medicine, Sechenov First Moscow State Medical University, 8-2 Trubetskaya Str., 119991 Moscow, Russia; 3 Interfaculty Research Centre on Biomaterials (CEIB), Chemistry Institute, University of Liège, B6C, 11 Allée du 6 Août, B-4000 Liege, Belgium; a.monteiro@uliege.be (A.P.F.M.); c.grandfils@uliege.be (C.G.); ange.ilangalabooka@uliege.be (A.B.I.)

**Keywords:** microparticles, regenerative medicine, biomaterials, tissue engineering, clinical application, emulsions, drug delivery, scaffolds

## Abstract

Tissue engineering and cell therapy are very attractive in terms of potential applications but remain quite challenging regarding the clinical aspects. Amongst the different strategies proposed to facilitate their implementation in clinical practices, biodegradable microparticles have shown promising outcomes with several advantages and potentialities. This critical review aims to establish a survey of the most relevant materials and processing techniques to prepare these micro vehicles. Special attention will be paid to their main potential applications, considering the regulatory constraints and the relative easiness to implement their production at an industrial level to better evaluate their application in clinical practices.

## 1. Introduction

**Figure 1:**
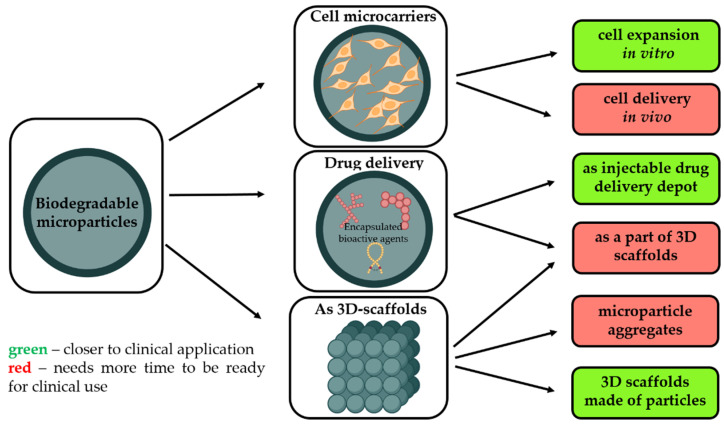
Different strategies of microparticles usage for regenerative medicine and perspective of their clinical application. Created by BioRender.com (accessed on 25 January 2022).

Regenerative medicine is one of the most attractive topics of research worldwide. Different strategies are proposed, and a range of materials of various forms and compositions tailored for tissue engineering are developed, but this approach just started to emerge in clinics [1]. This manuscript will provide an overview of the most relevant applications of biodegradable microparticles, which could more easily cross from the R&D stage to their implementation in the clinical reality while fitting the needs of regenerative medicine. As schematically presented in Figure 1, biodegradable microparticles (MPs) made from degradable and biocompatible polymers, with a mean diameter of ~200 μm, are attractive not only as 3D matrices to multiply cells but also as a scaffold to support tissue rebuilding [2]. Being injectable and made from safe materials, they could be implanted into the tissue defect adopting one of the three possible clinical approaches:

- As a temporary microcarrier to support expansion of cells initially cultivated in vitro [3]. Biodegradability is a crucial characteristic to avoid main technological issues related to cell multiplication on non-degradable microcarriers, i.e., poor yield of cell detachment, contamination related to enzymes requested to harvest cells, and difficulties to separate microparticle debris from free cells. Moreover, as these microcarriers are made from safe and degradable polymers, these cell microcarriers could be injected into the targeted tissues to restore them.
- Without pre-culture with cells in order to provide a sustained and local release of growth factors selected to promote tissue rebuilding while also offering a large surface to enhance in vivo cell adhesion.
- As a part of other types of 3D scaffolds for tissue engineering, including the application of biodegradable microparticles as starting building blocks to generate 3D scaffolds with well-defined architecture, adopting additive technologies or other techniques.

Despite the potentialities of these microparticles in tissue engineering today, up to our knowledge, there are no commercial biodegradable microcarrier-based products available on the market. However, it is worth reminding that the technologies requested to produce pharmaceutical-grade biodegradable microparticles have already been developed and validated for several applications in the pharmaceutical area. Indeed, these microparticles have been first designed to achieve a sustained release of (bio)pharmaceutical actives on an extended period [4]. Most of the difficulties of translation of all these R&D efforts and clinical trials on the application of biodegradable microparticles into the clinical reality come from several challenges and bottlenecks general issues of the cell-based products for regenerative medicine [5,6]. Indeed, several barriers have been identified, which can be classified according to technological, clinical, and administrative criteria.

Regarding the technological challenges, in comparison to classical drugs made from a single molecular entity, cells are a viable complex material that can change quickly in response to any subtle variations of their environment. Accordingly, the validation of a cell-based therapy product adopting the standards typically imposed in the pharmaceutical industry is impossible due to the intrinsic variation in the cell sampling and the difficulty of establishing standard reference products. The costs associated with the production, storage, and distribution of, for example, stem cells are today so expensive that their clinical use at a worldwide level remains mostly unrealistic [7]. Indeed, in contrast to classical molecular drugs, stem cells, once collected, purified, and multiplied, have to be either administrated just after or stored in liquid nitrogen to maintain their viability. The worldwide repartition of the ongoing clinical trials highlights that these biotherapies are limited to countries where harvesting cells or tissues of human origin can be performed in health centers under strict controls and accredited by Public Authorities.

A critical issue of cell therapy is the in vitro large-scale expansion of allogeneic cells according to Good Manufacturing Practice (GMP) standards which allows combining product quality, purity, reliability, and good yields [7]. In this perspective, biodegradable microparticles could be a better solution for the in vitro cultivation of substrate-dependent cells than 2D substrates. Mostly under the shape of plastic plates and T-flasks, these two surfaces are mainly coated by bioadhesive proteins such as Matrigel^®^ (Corning Inc., New York, NY, USA), gelatin, collagen, or even mouse embryonic fibroblasts (MEF) to enhance cell adhesion [8,9]. The total surface area required for cell expansion to satisfy a single therapeutic dose in the clinical assay is nearly 4.5 m^2^, corresponding to 600 standard T-75 culture plates. Nowadays, the most promising alternative approach relies on the cultivation of cells in suspension in closed and controlled stirred bioreactors in which the solid surface required for the anchorage of adherent cells is provided by microbeads suspended in the culture medium [8,9].

In clinical practice, the human cells as stem cells are collected mostly from critical tissues, such as bone marrow, umbilical cord, or fetal blood, where the recruitment of stem cell donors is difficult due to the invasive collection procedure. Recruitment of patients benefiting from these clinical trials is also a challenge due to the need to have a long-term follow-up. To date, if clinical trials are supporting that cell-based therapies are generally safe, their clinical benefits are still raising several concerns. For example, if Alofisel^®^ (Takeda Pharmaceutical Company Limited, Tokyo, Japan), a stem cell product designed for the treatment of complex perianal fistulas in adult Crohn’s patients, has been approved in 2018 by the European Medical Agency (EMA), cell therapy costs/clinical benefit ratio is too high, and the rationality of its clinical application is still under question [10]. Clinical assays have also demonstrated that cell therapies are not devoid of critical side effects. Indeed, immune rejection, embolization of blood vessels or lungs after cell administration as well as tumor induction, have been amongst others reported as main concerns with these new therapeutic approaches [6].

From the administration point of view, being novel for regulatory bodies, microparticles with pre-cultured cells impose more strict regulations which are regularly updated without harmonization between countries. This lack of standardization makes multicenter clinical studies more difficult. Additionally, the long-term monitoring requests to guarantee patient safety, and a lack of standardization in data collection and interpretation, represent additional major barriers. Accordingly, compared to typical clinical assays realized on classical drugs, clinical studies including cells are more expensive for all phases, and their duration is typically twice longer (3.5 to 4 years) [7].

Apart from these barriers in the practical employment of microcarriers, this review intends to report the various raw materials and technical approaches which have been adopted to optimize biodegradable microcarriers and to evaluate their in vitro/in vivo efficiency regarding the three abovementioned main applications, including the use as cell-seeded scaffolds.

## 2. Fabrication and Modification of Polymeric-Based Microparticles (MPs)

### 2.1. Materials for MPs Fabrication

There is a variety of materials that can be used for microparticles fabrication, as depicted in Table 1. Material selection should be made according to key technical features, such as degradation and diffusion features, thermal and mechanical properties, and suitable structural form for cell adhesion and tissue integration [11]. However, in terms of clinical application, the materials used for the microparticle fabrication should be safe and approved for biomedical use as the main criterion.

Biodegradable synthetic aliphatic polyesters are the main class of raw materials reported for the fabrication of polymeric scaffolds and drug delivery systems [11,12,13]. Being approved for more than 30 years by regulatory bodies, e.g., FDA or EMA, aliphatic polyesters, such as poly(lactic acid) (PLA), poly(glycolic acid) (PLGA), polycaprolactone (PCL), and their copolymers, have received the most considerable attention. The main requirements for their biomedical applications are biodegradability, tolerability, and non-toxicity. These polymers undergo hydrolytic degradation in the human body, releasing no cytotoxic byproducts. Thanks to the possibility to adjust their chemical structure, e.g., molecular weight, stereoregularity, macromolecule topology, copolymer composition, etc., these synthetic polymers are more reliable and flexible in terms of microparticle fabrication conditions and final properties. However, their lack of bioactivity, i.e., the inability to interact specifically with cells to promote and control cell adhesion, proliferation, and differentiation, limits their functionalities in tissue engineering. Thereby, to extend the functionality of microparticles based on synthetic polymers, they could be blended with natural polymers or inorganic components. Such types of microparticles are mainly interesting for the fabrication of microparticles without pre-cultured cells or as building blocks for additive technologies, i.e., selective laser sintering.

The main advantage offered by natural polymers relies on their ability to better mimic biological macromolecules and enhance specific cell adhesion. These materials are indeed more likely to stimulate cell attachment and proliferation due to specific integrin molecular recognition [14]. Their natural chemistry allows them to degrade through natural enzymatic pathways releasing no cytotoxic byproducts. However, being from natural sources, their purity level can raise several concerns in terms of reliability, homogeneity, stability, and potential immunogenicity [11].

Some polysaccharides and collagen are the most commonly studied natural polymers for microparticles processing [15]. Alginates are natural gel-forming polysaccharides of algal or bacterial origin and have been used in the food and pharmaceutical industries since 1881. In clinics, these polysaccharides are most specifically used daily as a material for wound dressings thanks to their hemostatic properties and biocompatibility. Alginate alone, or in combination with other natural polymers, has been mostly reported for cell encapsulation for several decades now [15,16,17,18,19]. However, due to their high hydrophilicity combined with a high negative charge density, alginates are suffering from the main drawback for tissue engineering, i.e., cell repulsion properties that counteract any cell adhesion and proliferation. For this reason, alginates functionalized with peptides with cell adhesion properties are today proposed for tissue engineering applications [20].

Chitosan is a linear polysaccharide produced via deacetylation of chitin (a primary component of cell walls in fungi, insects, and crustaceans). With a chemical structure similar to glycosaminoglycan, an important component of the human extracellular matrix and cell signaling pathways [21], its safety has been reported in several scientific papers. Furthermore, chitosan-based materials have antimicrobial properties and can stimulate angiogenesis [22]. However, till now, if some clinical trials have reported on the adoption of chitosan-based materials for tissue repair [23], these materials still need formal approval of legal authorities to be used in clinical practice.

Collagen is the most important protein of the extracellular matrix in the human body. Hence it has been widely adopted for tissue engineering applications [14,24]. Collagen shows high biocompatibility, low immunogenicity, cell adhesion, and proliferation. Products from its degradation absorb easily in the body and are not cytotoxic [25]. Collagen microparticles enable the delivery of both bioactive compounds and stem cells [26]. Gelatin, resulting from mild degradation of collagen, is also disclosed to be biodegradable and safe while forming gels very easily. Some studies have shown efficient cell growth and differentiation using gelatin hydrogel microspheres [16,27]. Further, gelatin is commonly used in combination with alginates in the form of hydrogels.

Polyanhydrides are a class of degradable synthetic biopolymers widely used as carriers for controlled drug delivery. Despite being easy and cheap to synthesize, polyanhydrides have a short shelf life. Thus, there are few polyanhydride products available on the market compared to polyester-based products, and a very low number of works explore their use in tissue engineering [28].

Polyhydroxyalkanoates (PHAs) are other biopolymers of bacterial nature produced under stress conditions as an energy reservoir. Such representatives, as homopolymer poly(3-hydroxybutyrate) or the poly(3-hydroxybutyrate-co-hydroxyvalerate) copolymer, may be efficiently used in biomedicine, e.g., drug delivery [29]. Their biocompatibility and biodegradability, along with good controllable mechanical properties, stimulate the research and allow to refer to PHAs as «green plastics» [30,31]. PHAs may promote cell growth and enable their adhesion and proliferation [31,32].

Silk fibroin has also been suggested as a material for microparticle fabrication. This natural protein derived from silkworms exhibits cell-binding moieties enabling stem cells differentiation [33]. Silk fibroin can be tailored to control its biodegradation and its mechanical strength [25]. This material can also contribute to drug delivery [34]. Despite high biocompatibility and several advantages, low mechanical strength, and batch-to-batch variability have been disclosed as the main limitations of these natural products for tissue engineering applications. In this perspective, at the present moment, synthetic polymers are more promising for the fabrication of microparticles for real clinical application. The novel research projects are mostly devoted to hybrid microparticles with the aim of combining the most beneficial properties of different materials. Synthetic materials, e.g., PLA, PGA, PCL, and their copolymers, contribute to their mechanical properties and controlled formation, whereas natural materials, e.g., chitosan, collagen, increase the microparticles’ compatibility with the native extracellular matrix. In this way, the limitations of each individual material can be overcome, and biomedical needs can be met. There are a few works dealing with the fabrication of microparticles based on copolymers of synthetic and natural polymers, which gives a combination of advantages of both polymer types as well as flexibility in terms of fabrication conditions [35,36,37]. However, such copolymers could be considered as new polymers and needed to be firstly approved for biomedical application, which is complicated from the regulatory point of view.

Inorganic components could also be used for microparticles fabrication, but are mostly used as filling materials and stabilizers for dispersions. Nevertheless, scientific works describe inorganic materials as the main building material for microparticles [38]. Some materials which are similar to natural components may mimic human tissue structure and therefore promote cell differentiation. For example, bone is a highly organized natural nanocomposite consisting of collagen fibers mineralized with hydroxyapatite nanocrystals [39]. Hence, the application of biodegradable microparticles containing hydroxyapatite (HA) is beneficial for bone regeneration.

**Table 1:** Biodegradable biomaterials for MPs fabrication.

Material	Chemical Nature, Crystallinity, Thermal Properties	Range of Degradation Rate and the Main Route of Degradation	Functionality	Advantages	Drawbacks	Approval Status	Ref.
Synthetic polymers							
PCL	Aliphatic polyester; Semi-crystalline; Tg: −60 °C; Tm: 60 °C	>1 year Ester hydrolysis	Hydrophobic material; Limited to aliphatic ester functions; Residual organic solvent content;	Macromolecular features and purity are well-controlled and reliable; Chemical purity is under control; Degradation rate can be easily adjusted in function of the Mw, tacticity, and crystallization %; Easy processability.	Lack of cell adhesion moieties; Release of acidic by-products during degradation.	FDA-approved	[40]
PLA	Aliphatic polyester; Semi-crystalline or amorphous; Tg: 40 °C; Tm: 180 °C	>0.6 year Ester hydrolysis					[41]
PLGA	Aliphatic polyester; Semi-crystalline or amorphous; Tg: 40 °C; Tm: 180 °C	>0.3 year Ester hydrolysis					[42]
Natural polymers							
Alginates	Anionic polysaccharides copolymers	Enzymatic degradation pathway	Carboxyl groups; Polyelectrolyte.	Gel-forming ability; Hydrophilicity.	No cell adhesion characteristics; Lack of control of the macromolecular features (Mw, polydispersity, purity).	FDA-approved	[43]
Collagen	Natural protein present in the extracellular matrices of tissues	Enzymatic degradation pathway	Carboxyl and amino groups	Cell adhesion and proliferation enhancement; Hydrophilicity.	Risk of allergic reactions; Low mechanical properties.	FDA-approved	[14]
Chitosan	Cationic polysaccharides copolymers.	Enzymatic degradation pathway	Primary amino-groups	Positive charge; Cell adhesion enhancement; Hydrophilicity.	Lack of control of the macromolecular features (Mw, polydispersity, purity); Difficulty of processing (not soluble in aqueous medium at neutral pH).	Not approved as pharmaceutical excipient; Under clinical testing as an implant.	[44]
PHAs	Polymers with high structural diversity; Semi-crystalline.	Enzymatic and hydrolytic degradation	Ester functions	Cell proliferation stimulation; Hydrophilicity; Controllable mechanical and thermal properties.	Low mechanical properties	Not approved	[45]
Silk fibroin	Natural protein isolated from animals.	Enzymatic degradation pathway	Carboxyl and amino groups	Cell proliferation stimulation; Hydrophilicity; Gel-forming material	High risk of allergic reactions.	Not approved	[46]

### 2.2. Methods of MPs Fabrication

**Figure 2:**
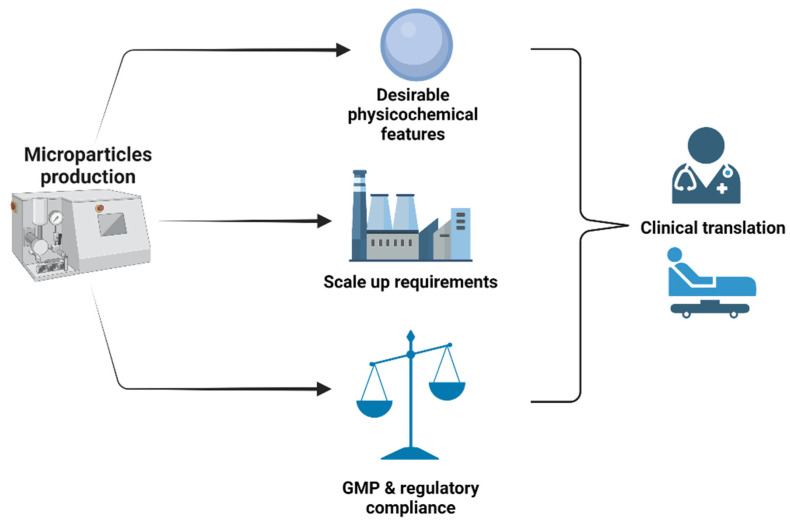
Schematic illustration representing the main three aspects to be taken under consideration while selecting a fabrication technology for microparticles. Created by BioRender.com (accessed on 23 February 2022).

Microparticles are well-established tools in pharmaceutical and biomedical fields with attractive applications, particularly in drug delivery and recently in regenerative medicine [29,47]. These particles provide several advantages, including the possibility of tailoring their properties to improve the efficiency of available biomedical applications that rely on them. They can be made from both degradable and non-degradable materials [48]. Over the last decades, successful developments in the polymers chemistry and processing field have catalyzed the design of microparticles with fine-tune characteristics. Currently, there is a wide range of techniques that have been developed for the production of microparticles [47,49,50,51,52]. The choice of the proper production method depends on such factors as particle composition, desired physical features, and optimal biochemical functionalities that need to be achieved with microparticles. Typically, the ideal manufacturing process should have control over critical microparticles features such as size, shape, surface topography, porosity, sustained and/or controlled release of encapsulated bioactive agents, and so forth. These characteristics are prerequisites for different applications, including cell therapy and tissue engineering, as they may impact cell attachment, spreading, morphology, and behavior during an in vitro expansion culture of cells or an in situ scaffold formation. The scalability of the process is also an important aspect that should be put under consideration. Indeed, commercial production of microparticles to be used for both pharmaceutical and cell products should comply with GMP conditions which require the use of fully characterized materials and well-defined equipment, as depicted in Figure 2. In addition to the plethora of conventional methods (emulsions, spray-drying, coacervation, etc.) that have been proposed to prepare microparticles, there is a remarkable research effort towards the development of innovative techniques capable of challenging many limitations encountered with conventional methods. This section will review both aspects.

#### 2.2.1. Emulsions

Emulsification is one of the most commonly used methods for polymeric microparticles production. In this technique, microparticles are obtained either by emulsion or double emulsion method where a solubilized polymer in an organic solvent is mixed with a larger volume of the aqueous phase in stirred tanks. This step is followed by washing, drying, and collecting the prepared microparticles [53]. Despite having facile handling with a good cost-effectiveness ratio, this technique is riddled with a couple of drawbacks related to its harsh conditions that may threaten the stability of fragile molecules (proteins, peptides, nucleic acids, etc.). Microparticles obtained using this technique often have a broad particle size distribution as shear forces are not spatially uniform throughout the processed batch [54]. Furthermore, there is a limited possibility to control critical physical features such as porosity, surface topography, and shape using this approach. To address these issues, several modifications have been made in the conventional emulsion method to obtain better outcomes. G.T. Vladisavljević et al. discussed in their work the use of membrane emulsification to produce structured microparticles with tailored properties for specific applications. In this method, emulsification is achieved by pressing a pure dispersed phase or a pre-emulsified mixture of the dispersed and continuous phase through a microporous membrane under controlled injection rate and shear conditions. Thus, the droplet size can be precisely controlled over a wide range, and narrow droplet size distribution can be obtained [55]. Naidoo et al. showed that adding a porogen agent such as sodium bicarbonate (NaHCO_3_) into the polymer oil phase and emulsification in an acidic polyvinyl alcohol (PVA) aqueous phase could help produce particles with a micro-porous hemi-shells structure. The particle size and displayed openings were about 50–200 µm and 20 µm, respectively [53]. Recently, Druel et al. suggested the emulsion-coagulation technique to prepare aerogel microparticles. Cellulose solution was added dropwise to paraffin oil and emulsified with a marine-style impeller. Drying was then carried out with supercritical CO_2_. The final results showed that spherical aerogel microparticles of a few tens of microns in diameter and with a high specific surface of approximately 350 m^2^/g were obtained [56].

The emulsification technique arouses interest as a simple and flexible method for producing more complex microparticles. One of the options is a composite MPs formation using various combinations of filling materials, polymers, etc. More complicated variations of the emulsion method, e.g., double emulsification and Pickering emulsion techniques, enable the fabrication of particles with an adjusted structure/morphology and therefore desired properties [57]. Possible loading with water-soluble molecules or unstable drugs is an important advantage compared to the single emulsion method [58], for instance, enclosing a drug-loaded hydrophilic core in a hydrophobic polymeric shell [59]. Porous silica materials are known to have a high drug-loading potential, and its encapsulation in biodegradable polymer PLGA via double emulsification method may help control and extend drug release where both components contribute to it [60,61]. Further, layered polymer-based microparticles engineered from PLGA with PLLA or PCL, respectively, via double emulsification may provide delayed predictable protein release, which is a promising feature in drug delivery [62]. This technique also allows step-by-step incorporation of functional additives, e.g., hydroxyapatite and silver nanoparticles, for introducing both cytocompatibility and antibacterial activity [63]. Shokrolahi et al. reported that HA particles might contribute to the osteogenic differentiation of stem cells as well as load and improve the release profile of active agents, playing a crucial role in regenerative medicine [64].

HA nanoparticles (nHA) dispersed in the aqueous phase also act as a stabilizer of the emulsion, which leads to core/shell MPs producing where the shell consists of a HA nanolayer [65]. This method is based on Pickering emulsion technology and allows to avoid common surfactant disadvantages such as cytotoxicity, hemolysis inducing, and low biocompatibility [66,67]. The irreversible adsorption and immobilization of solid nanoparticles on the oil-water interface is the main mechanism of Pickering stabilization [67]. Aside from hydroxyapatite, other solid molecules are used for both emulsification and improving biocompatibility as well as mechanical properties. In this perspective, chitosan particles appear as a great choice exhibiting acceleration of tissue regeneration and high biocompatibility combined with the stabilizing effect [68]. Another interesting example is when CaCO_3_ microparticles were used to stabilize alginate particles at the water/oil interface. This process induced gel formation, which resulted in an efficient protein encapsulation and release [69].

#### 2.2.2. Spray Drying

Spray-drying methods have been widely used for many years to produce microparticles [70]. The process consists of transforming a polymeric fluid material in dried microparticles, taking advantage of the fluid material atomization into a gaseous hot drying medium. Feed flow rate, inlet temperature, outlet temperature, atomization, drying gas type, and flow rate are amongst the main critical process parameters that impact the quality of final products [70]. Liquids of various types, such as emulsions and dispersions, can be converted into solid particles with controllable size, shape, and porosity. This technique provides many advantages, namely a rapid and simple process (high throughput), scalability, reproducibility, and cost-effectiveness [71,72]. It has also proven its suitability for the production of a wide variety of microparticle structures used as delivery carriers for various pharmacological active molecules, including proteins [73,74]. Manufacturing of nHA/chitosan composite microparticles via a spray-drying technique results in homogenous MPs mimicking bone structure with successfully incorporated nHA particles [75]. Another possibility is the formation of core/shell microparticles loaded with a dual bioactive encapsulation of incompatible agents. The process is performed using three or four fluid nozzles for incompatible biomaterials spraying, which enables the separation of them into core and shell layers [76]. The spray drying technique is also suitable for microparticle modification and surface coating, e.g., Lins et al. adjusted a chitosan coating on drug-loaded polyhydroxybutyrate-based MPs to obtain composite MPs with a sustainable drug release [77], and Silva et al. described shell layer formation with a drug encapsulation [78].

The major drawback of this technique is the degradation risk of encapsulated bioactive agents or organic components due to exposure to high temperatures. Even so, many studies report the production of complex microstructure (composites) using spray drying. Particularly, nano-in-micro-particles consisting of nano-polymeric particles embedded in microparticle matrices, such as chitosan, have been investigated for enhanced drug delivery and functional properties [75,79]. To minimize the impact of organic solvents and shear stress on the bioactive molecule’s condition, an electrodynamic spray-drying method may be implemented [80]. There is also data on the combination of a spray-drying technique and microfluidic approach, which is meant to produce more size- and structure-homogeneous particles with correlated properties [81].

#### 2.2.3. Microfluidics

Advances in different microfluidic devices (MD) have attracted much attention lately as they show the great power of this technology for the synthesis of micro-sized particles with unprecedented structure diversity [82]. The usefulness of MD is linked to their ability to finely control fluid and reaction conditions as the miniaturization associated with the large surface-to-volume ratio ensures a homogeneous reaction environment and efficient heat and mass transfer [83]. Such characteristics allow efficient control of kinetic parameters within a continuous flow regimen. The other advantages are better integration capability and reproducibility when compared to conventional bulk methods. Microfluidics has all the potential to meet the demands of the fabrication of uniform microparticles with flexible sizes, compositions (including multicompartmentalized microparticles), and internal structures [82], something very difficult to achieve by using other techniques such as precipitation, polymerization, and spray-drying. Microfluidics technology also enables the fabrication of spherical and non-spherical microparticles, core-shell MPs, Janus MPs, and other complex variations depending on the different approaches [82]. Janus particles (JPs) are composed of two or more materials, often of a different nature, whose unique properties are preserved and contribute to the whole microparticle [84]. In drug delivery, Janus particles may perform multi-drug loading and gradual drug release having multiple domains [85,86]. The big challenge about JPs is obtaining a desired size, composition, and shape, so a well-controlled fabrication method should be prioritized. Sun et al. produced PLGA-based JPs using a solvent evaporation-induced droplet phase separation technique assessed by a microfluidic chip that may help to avoid a spontaneous decomposition of JPs along with a strictly defined structure [86]. Moreover, microfluidics allows the generation of double emulsions in a two- or even single-step way [87] which may be more controllable and efficient than the emulsification technique. The fact that some parameters such as the fine-tune fluid dynamics, the surface or interface (hydrophilicity/lipophilicity/wettability) of the microfluidic devices, and the structure of the devices are now being easily implemented adjustably, the benefits of this remarkable technology will be further enlarged [82,88]. Based on these characteristics, microfluidic devices could be divided into 3 categories, namely chip-based, tubular microreactors, and centrifugal microreactors. Each of them has advantages and disadvantages (Table 2), and the choice of device depends broadly on the processing requirements and the nature of the product to produce. In a chip-based system, the flow transport, which is a key parameter, can be manipulated through variation in channel geometries (passive control). It is also possible to play with other inputs from external sources such as the electric field, magnetic field, optical force, and heat to tune the flow transport [88]. Microfluidic-based microreactors depart from laminar flow-based into turbulent flow processing to increase the efficiency of mixing and control the diffusion.

### 2.3. Microparticle-Based and Microparticle-Contained 3D Structures

Biodegradable microparticles are well-established tools in the pharmaceutical and biomedical fields with attractive applications. They are especially interesting as injectable drug/cell microcarriers, but they could also be successfully used for the fabrication of larger scaffolds using one of the three possible strategies:

- Aggregation of cell-free microparticles via polymer/polymer aggregation or assembly of microparticles with pre-cultured cells through cell/cell interactions;
- Microparticles as filling material to other types of matrices, including the application of them as drug depot, functional fillers to regulate the physico-mechanical properties of the matrix as well as cell-seeded microcarriers within bioinks;
- Microparticles without cells as building blocks for the fabrication of 3D scaffolds.

Aggregation of the biodegradable microparticles leading to the formation of larger objects could be realized using either chemical/physical interactions of microparticle surfaces or via interactions through cells seeded onto the microparticles [106]. The second approach is more interesting and consists of two stages, when (1) the microparticles used for cell expansion have their surface covered by cells, and (2) cell culture continues at milder conditions to promote cell-cell interactions [107]. This strategy of microparticle application needs significant control over MPs surface characteristics [108] and culture conditions [107]. Aggregation of the cell-laden microparticles could be carried out in vitro with an aim to form complex tissue/organ for further implantation, or cell-seeded microparticles could be firstly injected into the targeted tissue and agglomerate in situ by the action of the cells [109].

In the frame of the second strategy, the microparticles could be considered as a drug delivery depot [38,110], filling material to regulate properties of matrix [111,112,113], or as cell-seeded microcarriers for fabrication of complete scaffold combining polymeric matrix and cells [114]. The application of the microparticles as the bioactive component depot is discussed in the next section. Fabrication of larger scaffolds filled the microparticles are mainly used to enhance the physico-mechanical properties of the final material, such as mechanical characteristics, porosity, protein adsorption, mineralization, biocompatibility, etc. This concept of the microparticle application could be considered as one of the possible approaches to the fabrication of composite scaffolds. The requirements to the structure of microparticles used as filling materials are not so rigid since they rarely appear in contact with cells. Such “microfillers” are frequently made from naturally derived polymers and fabricated using mechanical destruction, e.g., milling [112]. As a more advanced approach, the biodegradable microparticles could be pre-seeded with cells before fabrication of a larger scaffold [115]. For example, filling bioinks with cell-laden microparticles allows to increase cell density within the bioink and to protect cells during bioprinting [115,116]. The clinical application of such cell-seeded microparticle-contained bioinks could be hardly expected in the near future as they should satisfy different technical specifications, regulatory rules as tissue-based products or as a degradable implant (class 3 medical device) [117].

The application of microparticles for the fabrication of 3D scaffolds using additive technologies is one of the actively studied topics in scientific literature. The third strategy, which employs microparticles for the fabrication of 3D scaffolds, relies on the formation of microparticles-based materials without cell pre-seeding, being realistic from a regulatory point of view. As a more simple approach, microparticles, i.e., polymeric powders, could be transformed into porous scaffolds via molding [118]. Such a technique provided limited control over scaffold porosity based on the variation of mean size, size distribution, and shape of the microparticles. On the other hand, additive technologies allow the manufacturing of microparticle-based scaffolds with a more well-defined architectonic. For example, selective laser sintering, a well-known 3D prototyping technique, relies on controlled laser radiation allowing sintering of thermoplastic polymeric microparticles into 3D structures [119]. This application of cell-free microparticles for tissue engineering requires much better control over particle characteristics than one needed in the case of filler proposes. Particle average size and size distribution must be adjusted in order to ensure a uniform bulk density during sintering and to achieve a 3D material with well-defined volume/surface characteristics. The adoption of spherical microparticles as a starting material for laser sintering provides a reduced volumetric shrinkage, which allows translating a digital model into a real 3D structure with high accuracy [120]. It is worth highlighting that composite microparticles are especially interesting for the fabrication of scaffolds for bone reconstruction in tissue engineering [121,122].

## 3. Biomedical Applications of MPs

### 3.1. Microparticles as Drug Delivery Depot to Promote Tissue Reconstruction

**Figure 3:**
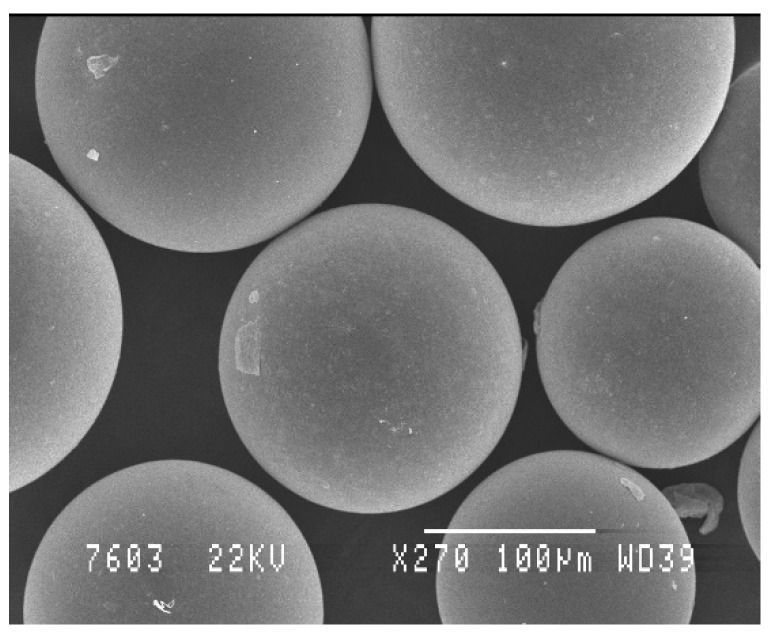
SEM micrographies of PLA microparticles prepared by oil-in-water emulsion process where growth factors can be loaded either as a solid dispersion in the oil, either adopting a double emulsion (W/O/W) evaporation procedure. SEM image of the microparticles was made by authors.

Those microparticles should be designed in order to guarantee the sustained release of growth factors for at least several days or weeks. Accordingly, and as given as an example in Figure 3, they should be essentially non-porous and made from polymers and according to a process that maintains the activity of these proteins during the whole duration of the release kinetics. Several growth factors have been proposed in the literature to enhance tissue repair and regeneration. Some of them, e.g., BMP-2 and BMP-6, are today accepted by legal authorities in order to assist bone reconstruction [123,124]. However, if the local delivery of these growth factors has highlighted promising results in pre-clinical studies, their success rate in clinic remains very limited [125]. Amongst the reasons explaining this lack of efficiency can be certainly mentioned the very short half-life of these biopharmaceutical agents, their rapid escape from the injected site, and also their high prices in comparison to their efficiency. These main limitations could be solved using degradable microparticles tailored to protect and progressively deliver one or a defined cocktail of growth factor(s) locally in the injured tissue. An additional advantage offered by this strategy will be that these microparticles will multiply the surface requested to support cell proliferation and differentiation. In terms of perspectives of valorization, this strategy also seems very attractive, taking into account the following aspects:

- Their local administration is minimally invasive and feasible into limited accessible sites;
- Their high surface/volume ratio is favorable and reported to be particularly suited as cell supporting microcarriers;
- Being made from well-known biocompatible and biodegradable polyesters, such as PLGA;
- Their degradation rate can be easily adjusted to balance growth factor release kinetics, cell supporting amplification, and mechanical support [107];
- The large scale GMP production of these drug delivery microparticles is already known and applied for several years;
- They are simple products, free of animal cells, and easy to submit to regulatory bodies;
- There is an opportunity to physically combine these microparticles with autologous stem cells just before implantation on a patient.

This approach was already suggested several years ago by Perez et al. [126] or J.P. Karam [127]. However, and despite all the advantages cited above highlighting the attractiveness of this approach, no microparticles are commercially available to trigger the sustained release of growth factors for tissue engineering.

Several reasons could be suggested to explain the reluctance of pharmaceutical industries to invest in this direction, in particular cost/efficiency. However, more importantly, supraphysiological concentrations of growth factors have been correlated with local and systemic adverse events, such as edema, tissue resorption, atypic remolding tissue, and also an increased risk of cancer development [128,129].

To counteract these issues and improve efficacy, chimeric growth factors have been recently developed and evaluated in vitro and in vivo for bone repair [130].

On the other hand, it is also worth reminding that tissue rebuilding is not only dependent on the presence of a specific growth factor, but is under the influence of several biological agents which regulate the proliferation, migration, and invasion of mesenchymal stem cells, according to a time sequence and concentration ratio which are not well defined [131].

Regarding these limitations, a concurrent strategy relies on the adoption of platelet concentrates which gain interest in clinical practice for both soft and hard tissue engineering. This autologous material, which does not require any specific regulatory requirements in the actual status of the legislation to be used by clinicians, allows a sustained release of concentrated growth factors as well as fibrin and platelets, and these compounds are known to be essential in wound healing [132].

### 3.2. Microcarriers for Cell Expansion

To be able to obtain a sufficient number of cells for cell therapy, whether it is about autologous or allogeneic stem cells, in vivo cell expansion is an essential step in the development process. Indeed, the final therapeutic dose of differentiated mesenchymal stem cells (MSC) varies according to application. It is reported that a therapeutic dose should contain between 2 and 3 million MSC/kg^−1^ body weight, bearing in mind that repeated doses are typically requested [133]. It is therefore not surprising that most indications have production requirements that cannot be met by traditional static tissue culture methods. In this context, the development of microparticle-based cell expansion technologies has gained momentum lately since they enable larger-scale production to ensure the continued progression of cell therapy through clinical trials [134,135].

Microcarriers are support matrices that enhance the growth of anchorage-dependent cells in bioreactor systems. In microcarrier (MC) culture, cells can grow as monolayers on the surface of small spheres but also as multilayers in the pores of microporous structures usually suspended in culture medium by gentle stirring. Since the introduction of this interesting approach by Van Wezel in 1967, several optimization works have followed, in particular, to improve the physicochemical properties of MC [136,137,138]. Those investigations have led to different microcarriers with varying surfaces, charges, structures, and other properties, allowing the customization of microcarriers’ surface properties based on cell types. They are now commercially available, although a significant proportion of the total investigated materials are still under development (Table 3).

**Figure 4:**
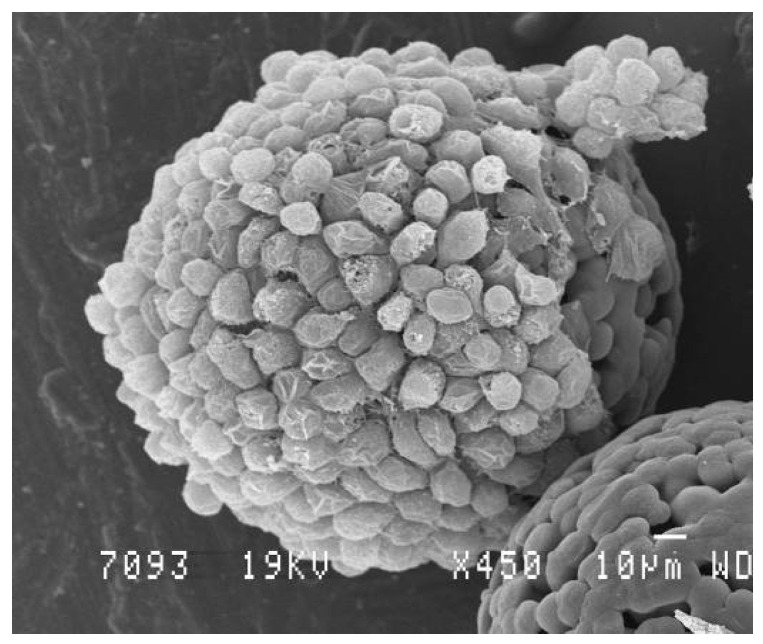
SEM micrography of degradable microparticles covered with fibroblasts L929 5 days after in vitro cell culture in DMEM medium at 37 °C. The surface of the microcarriers have been tailored to be rugous in order to promote cell adhesion. SEM image of the microparticles was made by authors.

There are some advantages with this manufacturing procedure which include the improvement of morphological aspects, mechanosensing properties, and cellular yield of the cultured cells [139]. The selection of a suitable microcarrier depends on the cell types and applications. Further engineering efforts are directed towards the design of MC with different formats that replicate signaling cues and the 3D network found in the native tissues of cells [138]. In this framework, MC surface features are key parameters that need to be controlled as they possess a huge impact on the cell fate [135,140]. As highlighted in Figure 4, the functionalization of the MC surface can be achieved through both physical and chemical means. Physical features of MC are related to topography, rugosity, stiffness, and elastic modulus, whereas chemical features include the use of various coating moieties. These characteristics are critical for attachment and detachment of cells to or from MC and may also dictate cells behavior (morphology, differentiation, biological functions, etc.). Cell culture on the microparticles could be additionally optimized in terms of dynamic culture conditions, i.e., stirring regime, microparticle/medium ratio.

**Figure 5:**
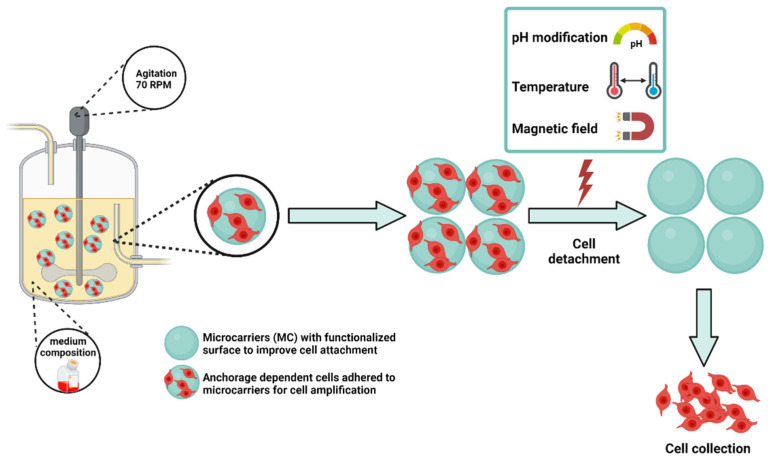
Culture system for scalable growth and controlled differentiation of stem cells using surface functionalized microcarriers (MC) to promote cell attachment. MC have stimuli responsive properties which can be used to trigger cell detachment, improving thereby the cells harvesting. Created by BioRender.com (accessed on 23rd February 2022).

Cell expansion carried out with non-degradable microcarriers still suffers from many drawbacks, for instance, poor yield of cell detachment, contamination issues related to proteolytic enzymes requested to harvest cells, and difficulties to separate microparticles debris from free cells [134]. Several strategies have been explored to improve harvest efficiency. Many of them rely on the use of stimuli-responsive polymers whose properties can be drastically altered either by a physical trigger (light, temperature, etc.) or a specific chemical reaction, as shown in Figure 5. For instance, poly N-isopropylacrylamide (PNIPAM) is one of the most studied thermoresponsive polymers used for its low critical solution temperature (LCST), which is in the range of 28–32 °C. It has been used for coating the surface of various MCs, including some commercially available ones, for thermal-induced cell detachment [141]. Recently, Narumi et al. assessed the use of a microcarrier coated with an innovative zwitterionic thermoresponsive polymer which had a lower critical solution temperature in the same range as PNIPAM for human mesenchymal stem cells growth. Compared to PNIPAM, this new polymer showed not only superior cell adhesion efficiency and growth rate, but also a higher cell recovery rate [142]. In another study, C. Li et al. reported alginate/PEG-based MC with cleavable cross-linkage for expansion and non-invasive harvest of human umbilical cord blood MSCs. Here, a contently cross-linked alginate network MC is degraded via cleaving of the S-S bonds using reductant, e.g., dithiothreitol. The cells harvested from this system had excellent viability and maintained the stemness and differentiation potential [143].

**Table 3:** Recent examples of polymeric microparticles used for cell expansion.

Fabrication Technology	Material Component (Matrix)	Types of Particles, Shape, and Dimension	Charge/Surface Area (cm^2^)/Density	Physicochemical and Biochemical Cues (Surface Coating)	Notes	Ref.
Emulsion solvent/evaporation	Poly (Ԑ-caprolactone)	Size between 224.5–366.3 μm	Pores size of 25.6–84.0 μm. Porosity of 57.4–75.5%.	Coating with hydroxyapatite	MC with surface modification supported very well the adhesion and growth of human fibroblasts.	[144]
Microinjection method	Alginate	Size of 421.94 μm. Spherical geometry	NA	Silk fibroin coating	Good adhesion of MSCs within 3 days of culture and preservation of their metabolic activity and multi-lineage differentiation of potential.	[145]
Needle/tubing microfluidic device	Polycaprolactone (80 KDa)	NA	NA	ECM coating (combination of fibronectin and poly-l-lysine)	Enhance attachment of human early MSCs at levels equivalent to the commercially available Cytadex 3MC. The cultured cells were able to induce bone formation in ectopic mouse model.	[146]
Electrospraying technique	Gelatin-Chitosan	Size of 350 μm	Density between 1.00–1.1 g/cm^3^. Pore size between 40 and 60 μm.	NA	Demonstration of the usefulness of gelatin-chitosan blends of different weight ratios as suitable material to prepare MC for supporting MSCs attachment and proliferation.	[147]
Emulsion-based thermally induced phase separation	Chitosan	Size of about 150 μm	Pores size varying from 20–50 μm.	NA	Excellent biocompatibility and unique pores’ structure, which allows hepatocyte culture in three-dimension space.	[148]
Cross-linked reaction	Gelatin	Size of about 250 μm	NA	NA	Report on cross-linked porous gelatin beads (redox-sensitive beads) that afford rapid, stimuli-triggered dissolution for facile cell removal of hMSC. Harvest time was reduced by at least 15-fold in a bioreactor of 3 L.	[149]
Emulsion solvent evaporation method	Polylactic-co-glycolic acid (PLGA)	Size about 260 μm	Negatively charged particles (−26.9 and−16.7 mV)	Poly L-Lysine (About 200,000 Dalton) Gelatin	Report on the development of US FDA MC that serves as an adherent platform for human umbilical vein endothelial cells (HUVEC). The cell density was multiplied up to 3.5 fold and healthy morphology.	[150]
W/O/W emulsion-based method	Poly-(ɣ-Benzyl-L-glutamate)	Size between 200–400 μm	Pores size above 50 μm	Janus microspheres	Open porous PBLG microcarriers with large pore size were prepared, demonstrating high cellular infiltration and proliferation rate of human adipose derived stem cells (hASCs)	[151]
Crystallization (organic solvent free process)	Poly (L-lactide) (PLLA) and poly (ethylene glycol) (PEG)	Size between 100–230 μm	NA	Functionalization with poly (L-ornithine), hyaluronic acid, and bioadhesive RGD peptide	hASCs were able to attach and grow on MCs whatever the surface treatment, but adhesion and proliferation were higher when the MCs were grafted with RGD	[152]

### 3.3. Microparticle-Containing 3D Scaffolds for Regenerative Medicine

Large scaffolds made of microparticles via the assembly of cell-free polymeric particles through various types of polymer/polymer interactions could be considered as a specific scaffold fabrication technique [153]. Aggregation of cell-seeded microparticles through the cell interactions is limited by issues coming from cell culture highlighted within previous sections and low control over the microparticle aggregation process.

An application of microparticles as an additional component of large 3D scaffolds refers to their usage as a drug depot, functional filling material, and cell-laden microcarriers. Works on the fabrication of scaffolds containing cell-seeded microparticles are mostly focused on technological issues. Indeed, this complex fabrication strategy is very promising but still requires a lot of processing optimization [115,154]. Thus, PLA microparticles functionalized with human recombinant collagen type I were seeded by mesenchymal stromal cells and cultured using either static or dynamic conditions before the loading of a bioink by the microparticles. The viability of cells seeded on the microparticles was monitored after the UV-induced crosslinking of the bioink made of gelatin methacrylamide. Microcarrier-laden bioink was successful in the fabrication of a composite scaffold. The application of the microparticles allowed to increase cell density in the bioink, preserve cell viability, and the microparticles also acted as mechanical reinforcement. Microparticles without preliminary cell culture could also be used as a part of a larger scaffold to regulate its properties (as functional filling material) or to provide sustainable release of bioactive compounds (as drug depot). In terms of biomedical application, the first approach is mostly aimed at the enhancement of biocompatibility of scaffolds made of synthetic polymers via filling them with naturally derived silk microparticles [112,113]. Such types of research are mainly focusing on bone tissue engineering, while the addition of microparticles as drug depots within larger scaffolds could be more diverse in terms of the final goal. Liu et al. reported that drug-loaded microparticles were successfully used as fillers within hydrogel inks for 3D extrusion printing of cartilage tissue scaffold [155]. Further, microparticles could be used as BMP depots to enhance osteogenesis [110] or as prolonged antibiotic release systems [17,155]. All of the above-mentioned strategies are based on the application of the microparticles as cell and/or drug microcarriers discussed in Section 3.1. and Section 3.2.

**Figure 6:**
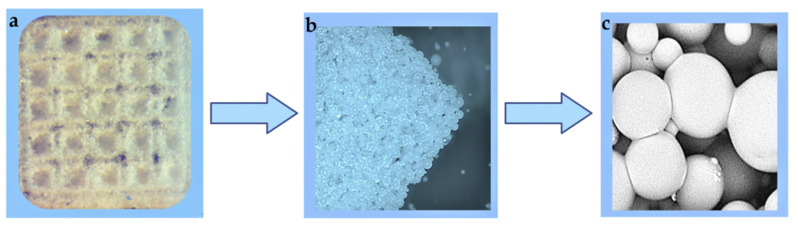
3D structures fabricated from polylactide microparticles via selective laser sintering: photo of the 3D scaffold (**a**), optical micrograph at higher magnification (**b**) and SEM image of the sintered microparticles (**c**) forming the 3D scaffold. Images of the 3D structures were made by authors.

Another promising approach is based on the fabrication of 3D structures with well-defined architectonics from cell-free microparticles. As shown in Figure 6, an application of the additive technologies allows for the manufacturing of scaffolds with desired structures, which are particularly interesting for bone tissue replacement. Indeed, native bone tissue has a complex structure both in terms of architecture and composition [156]. Still, additive technologies allow controlling scaffold’s 3D structure [157,158,159], while the usage of hybrid polymer/inorganic microparticles containing calcium phosphates mimic natural bone composition [160,161,162]. PCL powder was transformed into a porous scaffold with the aim of selective laser sintering technology, coated by collagen and used for chondrocytes culture [163]. This strategy allows for the fabrication of scaffolds with optimized microstructures and geometry per the needs of an individual patient.

## 4. Conclusions and Perspectives

Biodegradable microparticles, along with most of the other types of regenerative medicine tools, are very slowly entering into clinical practice. Several technological issues coming from the synthesis of safe and effective biodegradable materials as well as optimization of methods of microparticle fabrication are restraining the development of microparticles tailored for regenerative medicine. However, most of these problems could be solved at the present stage of scientific and technological development. Nevertheless, a wider and more complex range of criteria is preventing the clinical application of such microparticles in clinics. The clinical, technological, and administrative issues are mainly referring to economic reasons, a high cost of products, and administrative challenges coming from world heterogeneity.

Scientific literature is full of data and prospective results showing different and effective strategies of microparticle application in regenerative medicine. However, to bring these scientific findings closer to clinical reality, it would be useful to keep in mind all issues of future product valorization. We believe that cell expansion onto biodegradable microparticles and usage of cell-free particles for larger scaffold fabrication are two main approaches which are closer to clinical practice. Other strategies are promising, but they are more complex and need future optimization.

## Figures and Tables

**Table 2:** Summary of the MPs fabrication technologies.

Fabrication Technology	Critical Process Parameters	Advantages	Drawbacks	Scalability and GMP Compliance	Suitability for Cell Culture and Tissue Engineering	Ref.
Solvent extraction/evaporation based-Methods	Polymer concentration; Type and concentration of the stabilizer; Time and speed of homogenization; The ratio volume of dispersed and continuous phases.	Encapsulation of both hydrophilic and hydrophobic drugs (multiple W/O/W emulsion); Produces a wide range of particles size (from 10 to 300 μm); Cost effectiveness; Very well established and validated methods.	Poor particles size uniformity; Non control over shape, surface topography, and internal structure; High shear forces (degradation of shear sensitive ingredients); Multiple steps.	Kinam et al., reported recently a continuous in-line emulsification-extraction process capable of processing at flow rate of up to 400 mL/min to produce PLGA microparticles. This system can comply with GMP requirements.	Mesenchymal stem cells, adipose-derived stem cells, cardiac progenitor cells were successfully cultured and evaluated for various applications. All microparticles showed good biocompatibility.	[89,90,91,92]
Coacervation	Polymer type; Polymers ratio; pH; Ionic strength; Solvent evaporation rate.	Modulation of internal morphologies of microparticles; Controlled release kinetics of encapsulated agents.	Residual organic solvent content; Coacervating agents; Not suitable for size below 100 μm; Multiple steps.	Need to fully understand the impact of each process parameter for possible process scale up. This remains challenging.	Angiogenesis-inducing stem cell, mesenchymal stem cells were cultured for regenerative treatments.	[93,94,95,96]
Spray–drying	Feed flow rate; Inlet temperature; Outlet temperature; Atomization pressure; Type of drying gas; Flow rate.	Simple; High throughput; Size control; Reproducibility; Suitable for production of complex microstructures (composites).	Moderate yield for small batches; High temperature (degradation of heat sensitive compounds).	Attempts of scale-up have been carried out to produce functional microparticles for various pharmaceutical uses.	Cardiac stem cells, neonatal porcine sertoli cells, adrenal pheochromocytoma (P1C12) cells were either cultured or encapsulated into microparticles for various applications.	[97,98,99,100]
Membrane emulsification	Transmembrane pressure and flux; Shear stress; Membrane characteristics: surface wettability, charge, pore size, morphology, spatial arrangement, etc.; Formulation parameters: surfactant, viscosity, interfacial tension, etc.; Inject rate.	Process flexibility led to particles with versatile morphology (solid and hollow, matrix and core/shell, spherical and non-spherical, porous and coherent, composite and homogeneous; Tunable and narrow size distribution; High encapsulation efficiency of biocompounds (including proteins); Direct integration with downstream processing for further transformations of the formed droplets/particles.	Difficult to quantify the interplaying parameters (shear forces, interfacial tensions, etc.) that control the droplet size.	Large scale production can be carried out by transferring meaningful laboratory data for process scale-up. However, low emulsion throughputs and membrane fouling remain the main limitations for scale up.	Embryonic fibroblasts (NIH-3T3 cells), mesenchymal stem cells were investigated.	[55,101,102]
Microfluidic	Flow transport/flow rate; Geometry of microchannels; Surface or interface of the devices; External inputs (heat, light, magnetic field, etc.).	Efficient control of fluid and reaction conditions; Continuous flow operation; Achievement of tailored size, shapes, compositions, and internal structures of microparticles; Flexible drug encapsulation; High reproducibility; Direct integration with downstream processing for further transformations of the formed droplets/particles.	Dominance of surface forces; Relatively more expensive technology; Not fully automated; Cross contamination along the channel; Typically, low production rate (about 10 mL/h).	Parallelization of droplet generators in three-dimensional microfluidic devices has been widely proposed for large-scale production of microparticles. However, several challenges remain, especially in the development of systems that increase significantly fluid delivery while maintaining a uniform flow rate.	Mesenchymal stem cells were successfully expanded or encapsulated in various types of microparticles for tissue engineering constructs.	[103,104,105]

## Data Availability

Not applicable.

## References

[1] Hunsberger J.G., Shupe T., Atala A. (2018). An Industry-Driven Roadmap for Manufacturing in Regenerative Medicine. Stem Cells Transl. Med..

[2] Oliveira M.B., Mano J.F. (2011). Polymer-Based Microparticles in Tissue Engineering and Regenerative Medicine. Biotechnol. Prog..

[3] Derakhti S., Safiabadi-Tali S.H., Amoabediny G., Sheikhpour M. (2019). Attachment and Detachment Strategies in Microcarrier-Based Cell Culture Technology: A Comprehensive Review. Mater. Sci. Eng. C.

[4] Zhu M., Whittaker A.K., Han F.Y., Smith M.T. (2022). Journey to the Market: The Evolution of Biodegradable Drug Delivery Systems. Appl. Sci..

[5] Ratcliffe E., Thomas R.J., Williams D.J. (2011). Current Understanding and Challenges in Bioprocessing of Stem Cell-Based Therapies for Regenerative Medicine. Br. Med. Bull..

[6] Krause M., Phan T.G., Ma H., Sobey C.G., Lim R. (2019). Cell-Based Therapies for Stroke: Are We There Yet?. Front. Neurol..

[7] Laso-García F., Diekhorst L., Gómez-de Frutos M.C., Otero-Ortega L., Fuentes B., Ruiz-Ares G., Díez-Tejedor E., Gutiérrez-Fernández M. (2019). Cell-Based Therapies for Stroke: Promising Solution or Dead End? Mesenchymal Stem Cells and Comorbidities in Preclinical Stroke Research. Front. Neurol..

[8] McKee C., Chaudhry G.R. (2017). Advances and Challenges in Stem Cell Culture. Colloids Surf. B Biointerfaces.

[9] Chan S.W., Rizwan M., Yim E.K.F. (2020). Emerging Methods for Enhancing Pluripotent Stem Cell Expansion. Front. Cell Dev. Biol..

[10] Cossu G., Birchall M., Brown T., de Coppi P., Culme-Seymour E., Gibbon S., Hitchcock J., Mason C., Montgomery J., Morris S. (2018). Lancet Commission: Stem Cells and Regenerative Medicine. Lancet.

[11] Ozdil D., Aydin H.M. (2014). Polymers for Medical and Tissue Engineering Applications. J. Chem. Technol. Biotechnol..

[12] Molavi F., Barzegar-Jalali M., Hamishehkar H. (2020). Polyester Based Polymeric Nano and Microparticles for Pharmaceutical Purposes: A Review on Formulation Approaches. J. Control. Release.

[13] Demina T.S., Akopova T.A., Zelenetsky A.N. (2021). Materials Based on Chitosan and Polylactide: From Biodegradable Plastics to Tissue Engineering Constructions. Polym. Sci. Ser. C.

[14] Browne S., Zeugolis D.I., Pandit A. (2013). Collagen: Finding a Solution for the Source. Tissue Eng. Part A.

[15] Zakir Hossain K.M., Patel U., Ahmed I. (2014). Development of Microspheres for Biomedical Applications: A Review. Prog. Biomater..

[16] Yao R., Zhang R., Luan J., Lin F. (2012). Alginate and Alginate/Gelatin Microspheres for Human Adipose-Derived Stem Cell Encapsulation and Differentiation. Biofabrication.

[17] Yan J., Miao Y., Tan H., Zhou T., Ling Z., Chen Y., Xing X., Hu X. (2016). Injectable Alginate/Hydroxyapatite Gel Scaffold Combined with Gelatin Microspheres for Drug Delivery and Bone Tissue Engineering. Mater. Sci. Eng. C.

[18] Mahou R., Vlahos A.E., Shulman A., Sefton M.V. (2018). Interpenetrating Alginate-Collagen Polymer Network Microspheres for Modular Tissue Engineering. ACS Biomater. Sci. Eng..

[19] Bi Y.-G., Lin Z.-T., Deng S.-T. (2019). Fabrication and Characterization of Hydroxyapatite/Sodium Alginate/Chitosan Composite Microspheres for Drug Delivery and Bone Tissue Engineering. Mater. Sci. Eng. C.

[20] Lee K.Y., Mooney D.J. (2012). Alginate: Properties and Biomedical Applications. Prog. Polym. Sci..

[21] Francis Suh J.K., Matthew H.W.T. (2000). Application of Chitosan-Based Polysaccharide Biomaterials in Cartilage Tissue Engineering: A Review. Biomaterials.

[22] Sivashankari P.R., Prabaharan M. (2016). Prospects of Chitosan-Based Scaffolds for Growth Factor Release in Tissue Engineering. Int. J. Biol. Macromol..

[23] Mo X., Cen J., Gibson E., Wang R., Percival S.L. (2015). An Open Multicenter Comparative Randomized Clinical Study on Chitosan. Wound Repair Regen..

[24] Hong S., Hsu H.J., Kaunas R., Kameoka J. (2012). Collagen Microsphere Production on a Chip. Lab A Chip.

[25] Wang J., Sun X., Zhang Z., Wang Y., Huang C., Yang C., Liu L., Zhang Q. (2019). Silk Fibroin/Collagen/Hyaluronic Acid Scaffold Incorporating Pilose Antler Polypeptides Microspheres for Cartilage Tissue Engineering. Mater. Sci. Eng. C.

[26] Helary C., Browne S., Mathew A., Wang W., Pandit A. (2012). Transfection of Macrophages by Collagen Hollow Spheres Loaded with Polyplexes: A Step towards Modulating Inflammation. Acta Biomater..

[27] Hayashi K., Tabata Y. (2011). Preparation of Stem Cell Aggregates with Gelatin Microspheres to Enhance Biological Functions. Acta Biomater..

[28] Basu A., Domb A.J. (2018). Recent Advances in Polyanhydride Based Biomaterials. Adv. Mater..

[29] Campos E., Branquinho J., Carreira A.S., Carvalho A., Coimbra P., Ferreira P., Gil M.H.H., Campos E. (2013). Designing Polymeric Microparticles for Biomedical and Industrial Applications. Eur. Polym. J..

[30] Wei D.X., Dao J.W., Chen G.Q. (2018). A Micro-Ark for Cells: Highly Open Porous Polyhydroxyalkanoate Microspheres as Injectable Scaffolds for Tissue Regeneration. Adv. Mater..

[31] Ray S., Kalia V.C. (2017). Biomedical Applications of Polyhydroxyalkanoates. Indian J. Microbiol..

[32] Dwivedi R., Pandey R., Kumar S., Mehrotra D. (2020). Poly Hydroxyalkanoates (PHA): Role in Bone Scaffolds. J. Oral Biol. Craniofacial Res..

[33] Bhardwaj N., Rajkhowa R., Wang X., Devi D. (2015). Milled Non-Mulberry Silk Fibroin Microparticles as Biomaterial for Biomedical Applications. Int. J. Biol. Macromol..

[34] Mwangi T.K., Bowles R.D., Tainter D.M., Bell R.D., Kaplan D.L., Setton L.A. (2015). Synthesis and Characterization of Silk Fibroin Microparticles for Intra-Articular Drug Delivery. Int. J. Pharm..

[35] Demina T.S., Akopova T.A., Vladimirov L.V., Zelenetskii A.N., Markvicheva E.A., Grandfils C. (2016). Polylactide-Based Microspheres Prepared Using Solid-State Copolymerized Chitosan and D, L -Lactide. Mater. Sci. Eng. C.

[36] Yang L., Zhang J., He J., Zhang J., Gan Z. (2016). Fabrication, Hydrolysis and Cell Cultivation of Microspheres from Cellulose-Graft-Poly(l-Lactide) Copolymers. RSC Adv..

[37] Demina T.S., Drozdova M.G., Sevrin C., Compère P., Akopova T.A., Markvicheva E., Grandfils C. (2020). Biodegradable Cell Microcarriers Based on Chitosan/Polyester Graft-Copolymers. Molecules.

[38] Fu H., Rahaman M.N., Brown R.F., Day D.E. (2013). Evaluation of BSA Protein Release from Hollow Hydroxyapatite Microspheres into PEG Hydrogel. Mater. Sci. Eng. C Mater. Biol. Appl..

[39] Silva G.A., Coutinho O.P., Ducheyne P., Reis R.L. (2007). Materials in Particulate Form for Tissue Engineering. 2. Applications in Bone. J. Tissue Eng. Regen. Med..

[40] Dwivedi R., Kumar S., Pandey R., Mahajan A., Nandana D., Katti D.S., Mehrotra D. (2020). Polycaprolactone as Biomaterial for Bone Scaffolds: Review of Literature. J. Oral Biol. Craniofacial Res..

[41] Im S.H., Im D.H., Park S.J., Chung J.J., Jung Y., Kim S.H. (2021). Stereocomplex Polylactide for Drug Delivery and Biomedical Applications: A Review. Molecules.

[42] Cipurković A., Horozić E., Đonlagić N., Marić S., Saletović M., Ademović Z. (2018). Biodegradable Polymers: Production, Properties and Application in Medicine. Technol. Acta Sci./Prof. J. Chem. Technol..

[43] Fernando I.P.S., Lee W.W., Han E.J., Ahn G. (2020). Alginate-Based Nanomaterials: Fabrication Techniques, Properties, and Applications. Chem. Eng. J..

[44] Ahsan S., Bhatnagar I. (2018). Chitosan as Biomaterial in Drug Delivery and Tissue Engineering. Int. J. Biol. Macromol..

[45] Grigore M.E., Grigorescu R.M., Iancu L., Ion R.M., Zaharia C., Andrei E.R. (2019). Methods of Synthesis, Properties and Biomedical Applications of Polyhydroxyalkanoates: A Review. J. Biomater. Sci..

[46] Melke J., Midha S., Ghosh S., Ito K., Hofmann S. (2016). Silk Fibroin as Biomaterial for Bone Tissue Engineering. Acta Biomater..

[47] Lengyel M., Kállai-Szabó N., Antal V., Laki A.J., Antal I. (2019). Microparticles, Microspheres, and Microcapsules for Advanced Drug Delivery. Sci. Pharm..

[48] da Costa R.C., Pereira E.D., Silva F.M., de Jesus E.O., Souza F.G. (2018). Drug Micro-Carriers Based on Polymers and Their Sterilization. Chem. Chem. Technol..

[49] Meng F., Jiang Y., Sun Z., Yin Y., Li Y. (2009). Electrohydrodynamic Liquid Atomization of Biodegradable Polymer Microparticles: Effect of Electrohydrodynamic Liquid Atomization Variables on Microparticles. J. Appl. Polym. Sci..

[50] Morais A.Í.S., Vieira E.G., Afewerki S., Sousa R.B., Honorio L.M.C., Cambrussi A.N.C.O., Santos J.A., Bezerra R.D.S., Furtini J.A.O., Silva-Filho E.C. (2020). Fabrication of Polymeric Microparticles by Electrospray: The Impact of Experimental Parameters. J. Funct. Biomater..

[51] Tasci M.E., Dede B., Tabak E., Gur A., Sulutas R.B., Cesur S., Ilhan E., Lin C.C., Paik P., Ficai D. (2021). Production, Optimization and Characterization of Polylactic Acid Microparticles Using Electrospray with Porous Structure. Appl. Sci..

[52] Bhujel R., Maharjan R., Kim N.A., Jeong S.H. (2021). Practical Quality Attributes of Polymeric Microparticles with Current Understanding and Future Perspectives. J. Drug Deliv. Sci. Technol..

[53] Naidoo K., Rolfes H., Easton K., Moolman S., Chetty A., Richter W., Nilen R. (2008). An Emulsion Preparation for Novel Micro-Porous Polymeric Hemi-Shells. Mater. Lett..

[54] Li B., Wang X., Wang Y., Gou W., Yuan X., Peng J., Guo Q., Lu S. (2015). Past, Present, and Future of Microcarrier-Based Tissue Engineering. J. Orthop. Transl..

[55] Vladisavljević G.T. (2015). Structured Microparticles with Tailored Properties Produced by Membrane Emulsification. Adv. Colloid Interface Sci..

[56] Druel L., Kenkel A., Baudron V., Buwalda S., Budtova T. (2020). Cellulose Aerogel Microparticles via Emulsion-Coagulation Technique. Biomacromolecules.

[57] Lee Y.S., Johnson P.J., Robbins P.T., Bridson R.H. (2013). Production of Nanoparticles-in-Microparticles by a Double Emulsion Method: A Comprehensive Study. Eur. J. Pharm. Biopharm..

[58] Giri T.K., Choudhary C., Ajazuddin, Alexander A., Badwaik H., Tripathi D.K. (2013). Prospects of Pharmaceuticals and Biopharmaceuticals Loaded Microparticles Prepared by Double Emulsion Technique for Controlled Delivery. Saudi Pharm. J..

[59] Pacheco D.P., Amaral M.H., Reis R.L., Marques A.P., Correlo V.M. (2015). Development of an Injectable PHBV Microparticles-GG Hydrogel Hybrid System for Regenerative Medicine. Int. J. Pharm..

[60] Nan K., Ma F., Hou H., Freeman W.R., Sailor M.J., Cheng L. (2014). Porous Silicon Oxide–PLGA Composite Microspheres for Sustained Ocular Delivery of Daunorubicin. Acta Biomater..

[61] Nanaki S., Siafaka P.I., Zachariadou D., Nerantzaki M., Giliopoulos D.J., Triantafyllidis K.S., Kostoglou M., Nikolakaki E., Bikiaris D.N. (2017). PLGA/SBA-15 Mesoporous Silica Composite Microparticles Loaded with Paclitaxel for Local Chemotherapy. Eur. J. Pharm. Sci..

[62] Dutta D., Fauer C., Hickey K., Salifu M., Stabenfeldt S.E. (2017). Tunable Delayed Controlled Release Profile from Layered Polymeric Microparticles. J. Mater. Chemistry. B Mater. Biol. Med..

[63] Zhou Q., Wang T., Wang C., Wang Z., Yang Y., Li P., Cai R., Sun M., Yuan H., Nie L. (2020). Synthesis and Characterization of Silver Nanoparticles-Doped Hydroxyapatite/Alginate Microparticles with Promising Cytocompatibility and Antibacterial Properties. Colloids Surf. A Physicochem. Eng. Asp..

[64] Shokrolahi F., Khodabakhshi K., Shokrollahi P., Badiani R., Moghadam Z.M. (2019). Atorvastatin Loaded PLGA Microspheres: Preparation, HAp Coating, Drug Release and Effect on Osteogenic Differentiation of ADMSCs. Int. J. Pharm..

[65] Fujii S., Okada M., Sawa H., Furuzono T., Nakamura Y. (2009). Hydroxyapatite Nanoparticles as Particulate Emulsifier: Fabrication of Hydroxyapatite-Coated Biodegradable Microspheres. Langmuir.

[66] Chevalier Y., Bolzinger M.A. (2013). Emulsions Stabilized with Solid Nanoparticles: Pickering Emulsions. Colloids Surf. A Physicochem. Eng. Asp..

[67] ben Cheikh F., Mabrouk A.B., Magnin A., Putaux J.-L., Boufi S. (2021). Chitin Nanocrystals as Pickering Stabilizer for O/W Emulsions: Effect of the Oil Chemical Structure on the Emulsion Properties. Colloids Surf. B Biointerfaces.

[68] Asfour M.H., Elmotasem H., Mostafa D.M., Salama A.A.A. (2017). Chitosan Based Pickering Emulsion as a Promising Approach for Topical Application of Rutin in a Solubilized Form Intended for Wound Healing: In Vitro and in Vivo Study. Int. J. Pharm..

[69] Xu W., Zhu D., Li Z., Luo D., Hang L., Jing J., Shah B.R. (2019). Controlled Release of Lysozyme Based Core/Shells Structured Alginate Beads with CaCO3 Microparticles Using Pickering Emulsion Template and in Situ Gelation. Colloids Surf. B: Biointerfaces.

[70] Barra P.A., Márquez K., Gil-Castell O., Mujica J., Ribes-Greu A., Faccini M. (2019). Spray-Drying Performance and Thermal Stability Of. Molecules.

[71] Encina C., Márquez-Ruiz G., Holgado F., Giménez B., Vergara C., Robert P. (2018). Effect of Spray-Drying with Organic Solvents on the Encapsulation, Release and Stability of Fish Oil. Food Chem..

[72] Gonçalves A., Estevinho B.N., Rocha F. (2022). Spray-Drying of Oil-in-Water Emulsions for Encapsulation of Retinoic Acid: Polysaccharide- and Protein-Based Microparticles Characterization and Controlled Release Studies. Food Hydrocoll..

[73] Wu C., van de Weert M., Baldursdottir S.G., Yang M., Mu H. (2018). Effect of Excipients on Encapsulation and Release of Insulin from Spray-Dried Solid Lipid Microparticles. Int. J. Pharm..

[74] Carlan I.C., Estevinho B.N., Rocha F. (2020). Production of Vitamin B1 Microparticles by a Spray Drying Process Using Different Biopolymers as Wall Materials. Can. J. Chem. Eng..

[75] Ruphuy G., Saralegi A., Lopes J.C., Dias M.M., Barreiro M.F. (2016). Spray Drying as a Viable Process to Produce Nano-Hydroxyapatite/Chitosan (n-HAp/CS) Hybrid Microparticles Mimicking Bone Composition. Adv. Powder Technol..

[76] Gover Antoniraj M., Maria Leena M., Moses J.A., Anandharamakrishnan C. (2020). Cross-Linked Chitosan Microparticles Preparation by Modified Three Fluid Nozzle Spray Drying Approach. Int. J. Biol. Macromol..

[77] Lins L.C., Bazzo G.C., Barreto P.L.M., Pires A.T.N. (2014). Composite PHB/Chitosan Microparticles Obtained by Spray Drying: Effect of Chitosan Concentration and Crosslinking Agents on Drug Relesase. Artic. J. Braz. Chem. Soc..

[78] Silva D.M., Liu R., Gonçalves A.F., da Costa A., Castro Gomes A., Machado R., Vongsvivut J., Tobin M.J., Sencadas V. (2021). Design of Polymeric Core-Shell Carriers for Combination Therapies. J. Colloid Interface Sci..

[79] Spindler L.M., Feuerhake A., Ladel S., Günday C., Flamm J., Günday-Türeli N., Türeli E., Tovar G.E.M., Schindowski K., Gruber-Traub C. (2021). Nano-in-Micro-Particles Consisting of PLGA Nanoparticles Embedded in Chitosan Microparticles via Spray-Drying Enhances Their Uptake in the Olfactory Mucosa. Front. Pharmacol..

[80] Bizeau J., Mertz D. (2021). Design and Applications of Protein Delivery Systems in Nanomedicine and Tissue Engineering. Adv. Colloid Interface Sci..

[81] Liu W., Chen X.D., Selomulya C. (2015). On the Spray Drying of Uniform Functional Microparticles. Particuology.

[82] Jo Y.K., Lee D. (2020). Biopolymer Microparticles Prepared by Microfluidics for Biomedical Applications. Small.

[83] Xia H., Li J., Man J., Man L., Zhang S., Li J. (2021). Recent Progress in Preparation of Functional Microparticles Based on Microfluidic Technique. Mater. Today Commun..

[84] Su H., Hurd Price C.A., Jing L., Tian Q., Liu J., Qian K. (2019). Janus Particles: Design, Preparation, and Biomedical Applications. Mater. Today Bio.

[85] Zhang L., Zhang M., Zhou L., Han Q., Chen X., Li S., Li L., Su Z., Wang C. (2018). Dual Drug Delivery and Sequential Release by Amphiphilic Janus Nanoparticles for Liver Cancer Theranostics. Biomaterials.

[86] Sun X.T., Guo R., Wang D.N., Wei Y.Y., Yang C.G., Xu Z.R. (2019). Microfluidic Preparation of Polymer-Lipid Janus Microparticles with Staged Drug Release Property. J. Colloid Interface Sci..

[87] Wang J.-T., Wang J., Han J.-J. (2011). Fabrication of Advanced Particles and Particle-Based Materials Assisted by Droplet-Based Microfluidics. Small.

[88] Luo X., Su P., Zhang W., Raston C.L. (2019). Microfluidic Devices in Fabricating Nano or Micromaterials for Biomedical Applications. Adv. Mater. Technol..

[89] Freitas S., Merkle H.P., Gander B. (2005). Microencapsulation by Solvent Extraction/Evaporation: Reviewing the State of the Art of Microsphere Preparation Process Technology. J. Control. Release.

[90] Zhang J., Wang J., Qiao F., Liu Y., Zhou Y., Li M., Ai M., Yang Y., Sui L., Zhou Z. (2021). Polymeric Non-Spherical Coarse Microparticles Fabricated by Double Emulsion-Solvent Evaporation for Simvastatin Delivery. Colloids Surf. B Biointerfaces.

[91] Satapathy S.R., Sahoo R.N., Satapathy B., Immani R., Panigrahi L., Mallick S. (2021). Development and Characterization of Leuprolide Acetate Encapsulated Plga Microspheres for Parenteral Controlled Release Depot Injection. Indian J. Pharm. Educ. Res..

[92] Panigrahi D., Sahu P.K., Swain S., Verma R.K. (2021). Quality by Design Prospects of Pharmaceuticals Application of Double Emulsion Method for PLGA Loaded Nanoparticles. SN Appl. Sci..

[93] Kang M.K., Dai J., Kim J.C. (2012). Ethylcellulose Microparticles Containing Chitosan and Gelatin: PH-Dependent Release Caused by Complex Coacervation. J. Ind. Eng. Chem..

[94] Ach D., Briançon S., Broze G., Puel F., Rivoire A., Galvan J.M., Chevalier Y. (2015). Formation of Microcapsules by Complex Coacervation. Can. J. Chem. Eng..

[95] Scalera F., Gervaso F., de Benedictis V.M., Madaghiele M., Demitri C. (2016). Synthesis of Chitosan-Based Sub-Micrometric Particles by Simple Coacervation. IEEE Trans. Nanotechnol..

[96] Bastos L.P.H., de Sá Costa B., Siqueira R.P., Garcia-Rojas E.E. (2020). Complex Coacervates of β-Lactoglobulin/Sodium Alginate for the Microencapsulation of Black Pepper (Piper Nigrum L.) Essential Oil: Simulated Gastrointestinal Conditions and Modeling Release Kinetics. Int. J. Biol. Macromol..

[97] Giovagnoli S., Mancuso F., Vannini S., Calvitti M., Piroddi M., Pietrella D., Arato I., Falabella G., Galli F., Moretti M. (2014). Microparticle-Loaded Neonatal Porcine Sertoli Cells for Cell-Based Therapeutic and Drug Delivery System. J. Control. Release.

[98] Zhou P., Wu J., Xia Y., Yuan Y., Zhang H., Xu S., Lin K. (2018). Loading BMP-2 on Nanostructured Hydroxyapatite Microspheres for Rapid Bone Regeneration. Int. J. Nanomed..

[99] Soni G., Yadav K.S., Gupta M.K. (2020). QbD Based Approach for Formulation Development of Spray Dried Microparticles of Erlotinib Hydrochloride for Sustained Release. J. Drug Deliv. Sci. Technol..

[100] Joshi D.J., Chitre N.M., Bansal A., Murnane K.S., D’Souza M.J. (2021). Formulation and Characterization of Microcapsules Encapsulating PC12 Cells as a Prospective Treatment Approach for Parkinson’s Disease. AAPS PharmSciTech.

[101] Piacentini E., Lakshmi D.S., Figoli A., Drioli E., Giorno L. (2013). Polymeric Microspheres Preparation by Membrane Emulsification-Phase Separation Induced Process. J. Membr. Sci..

[102] Piacentini E., Dragosavac M., Giorno L. (2018). Pharmaceutical Particles Design by Membrane Emulsification: Preparation Methods and Applications in Drug Delivery. Curr. Pharm. Des..

[103] Zhang M.J., Zhang P., Qiu L.D., Chen T., Wang W., Chu L.Y. (2020). Controllable Microfluidic Fabrication of Microstructured Functional Materials. Biomicrofluidics.

[104] Liu Z., Fontana F., Python A., Hirvonen J.T., Santos H.A. (2020). Microfluidics for Production of Particles: Mechanism, Methodology, and Applications. Small.

[105] Lee S., de Rutte J., Dimatteo R., Koo D., di Carlo D. (2021). Scalable Fabrication and Use of 3D Structured Microparticles Spatially Functionalized with Biomolecules. ACS Nano.

[106] Ahrens C.C., Dong Z., Li W. (2017). Engineering Cell Aggregates through Incorporated Polymeric Microparticles. Acta Biomater..

[107] Simitzi C., Vlahovic M., Georgiou A., Keskin-Erdogan Z., Miller J., Day R.M. (2020). Modular Orthopaedic Tissue Engineering With Implantable Microcarriers and Canine Adipose-Derived Mesenchymal Stromal Cells. Front. Bioeng. Biotechnol..

[108] Privalova A., Markvicheva E., Sevrin C., Drozdova M., Kottgen C., Gilbert B., Ortiz M., Grandfils C. (2015). Biodegradable Polyester-Based Microcarriers with Modified Surface Tailored for Tissue Engineering. J. Biomed. Mater. Res.-Part A.

[109] García Cruz D.M., Sardinha V., Escobar Ivirico J.L., Mano J.F., Gómez Ribelles J.L. (2013). Gelatin Microparticles Aggregates as Three-Dimensional Scaffolding System in Cartilage Engineering. J. Mater. Sci. Mater. Med..

[110] Quinlan E., López-Noriega A., Thompson E., Kelly H.M., Cryan S.A., O’Brien F.J. (2015). Development of Collagen–Hydroxyapatite Scaffolds Incorporating PLGA and Alginate Microparticles for the Controlled Delivery of RhBMP-2 for Bone Tissue Engineering. J. Control. Release.

[111] DeVolder R.J., Kim I.W., Kim E.-S., Kong H. (2012). Modulating the Rigidity and Mineralization of Collagen Gels Using Poly(Lactic-Co-Glycolic Acid) Microparticles. Tissue Eng. Part A.

[112] Bhagabati P., Bhasney S.M., Bose D., Remadevi R., Setty M., Rajkhowa R., Katiyar V. (2020). Silk and Wool Protein Microparticle-Reinforced Crystalline Polylactic Acid Biocomposites with Improved Cell Interaction for Targeted Biomedical Applications. ACS Appl. Polym. Mater..

[113] Vyas C., Zhang J., Øvrebø Ø., Huang B., Roberts I., Setty M., Allardyce B., Haugen H., Rajkhowa R., Bartolo P. (2021). 3D Printing of Silk Microparticle Reinforced Polycaprolactone Scaffolds for Tissue Engineering Applications. Mater. Sci. Eng. C.

[114] Xu Y., Peng J., Richards G., Lu S., Eglin D. (2019). Optimization of Electrospray Fabrication of Stem Cell–Embedded Alginate–Gelatin Microspheres and Their Assembly in 3D-Printed Poly(ε-Caprolactone) Scaffold for Cartilage Tissue Engineering. J. Orthop. Transl..

[115] Levato R., Visser J., Planell J.A., Engel E., Malda J., Mateos-Timoneda M.A. (2014). Biofabrication of Tissue Constructs by 3D Bioprinting of Cell-Laden Microcarriers. Biofabrication.

[116] Leberfinger A.N., Ravnic D.J., Dhawan A., Ozbolat I.T. (2017). Concise Review: Bioprinting of Stem Cells for Transplantable Tissue Fabrication. Stem Cells Transl. Med..

[117] Oberweis C.V., Marchal J.A., López-Ruiz E., Gálvez-Martín P. (2020). A Worldwide Overview of Regulatory Frameworks for Tissue-Based Products. Tissue Eng. Part B Rev..

[118] Du L., Yang S., Li W., Li H., Feng S., Zeng R., Yu B., Xiao L., Nie H.-Y., Tu M. (2017). Scaffold Composed of Porous Vancomycin-Loaded Poly(Lactide- Co -Glycolide) Microspheres: A Controlled-Release Drug Delivery System with Shape-Memory Effect. Mater. Sci. Eng. C.

[119] Yuan S., Shen F., Chua C.K., Zhou K. (2019). Polymeric Composites for Powder-Based Additive Manufacturing: Materials and Applications. Prog. Polym. Sci..

[120] Mazzoli A. (2013). Selective Laser Sintering in Biomedical Engineering. Med. Biol. Eng. Comput..

[121] Yan D., Zeng B., Han Y., Dai H., Liu J., Sun Y., Li F. (2020). Preparation and Laser Powder Bed Fusion of Composite Microspheres Consisting of Poly(Lactic Acid) and Nano-Hydroxyapatite. Addit. Manuf..

[122] Krokos A., Gazinska M., Kryszak B., Dzienny P., Stepak B., Olejarczyk M., Gruber P., Kwiatkowski R., Bondyra A., Antonczak A. (2020). Comparison of Thermal, Structural and Morphological Properties of Poly(L-Lactide) and Poly(L-Lactide)/Hydroxyapatite Microspheres for Laser Sintering Processes. Polimery.

[123] McKay W.F., Peckham S.M., Badura J.M. (2007). A Comprehensive Clinical Review of Recombinant Human Bone Morphogenetic Protein-2 (INFUSE^®^ Bone Graft). Int. Orthop..

[124] Dimar J.R., Glassman S.D., Burkus J.K., Pryor P.W., Hardacker J.W., Carreon L.Y. (2009). Clinical and Radiographic Analysis of an Optimized RhBMP-2 Formulation as an Autograft Replacement in Posterolateral Lumbar Spine Arthrodesis. J. Bone Jt. Surg.-Ser. A.

[125] Ren X., Zhao M., Lash B., Martino M.M., Julier Z. (2020). Growth Factor Engineering Strategies for Regenerative Medicine Applications. Front. Bioeng. Biotechnol..

[126] Perez R.A., El-Fiqi A., Park J.H., Kim T.H., Kim J.H., Kim H.W. (2014). Therapeutic Bioactive Microcarriers: Co-Delivery of Growth Factors and Stem Cells for Bone Tissue Engineering. Acta Biomater..

[127] Karam J. (2014). Development of Pharmacologically Active Microcarriers Transporting Stem Cells and Releasing Growth Factors for Cardiac Tissue-Engineering. Ph.D. Thesis.

[128] Barcak E.A., Beebe M.J. (2017). Bone Morphogenetic Protein: Is There Still a Role in Orthopedic Trauma in 2017?. Orthop. Clin. North Am..

[129] Krishnakumar G.S., Roffi A., Reale D., Kon E., Filardo G. (2017). Clinical Application of Bone Morphogenetic Proteins for Bone Healing: A Systematic Review. Int. Orthop..

[130] Seeherman H.J., Berasi S.P., Brown C.T., Martinez R.X., Sean Juo Z., Jelinsky S., Cain M.J., Grode J., Tumelty K.E., Bohner M. (2019). A BMP/Activin A Chimera Is Superior to Native BMPs and Induces Bone Repair in Nonhuman Primates When Delivered in a Composite Matrix. Sci. Transl. Med..

[131] Somers P., Cornelissen R., Thierens H., van Nooten G. (2012). An Optimized Growth Factor Cocktail for Ovine Mesenchymal Stem Cells. Growth Factors.

[132] Ding Z.-Y., Tan Y., Peng Q., Zuo J., Li N. (2021). Novel Applications of Platelet Concentrates in Tissue Regeneration (Review). Exp. Ther. Med..

[133] Trounson A., McDonald C. (2015). Stem Cell Therapies in Clinical Trials: Progress and Challenges. Cell Stem Cell.

[134] Insights G.T., Therapy G., Rafiq Q.A. (2016). Toward a Scalable and Consistent Manufacturing Process for the Production of Human MSCs. Cell Gene Ther. Insights.

[135] Tavassoli H., Alhosseini S.N., Tay A., Chan P.P.Y., Weng Oh S.K., Warkiani M.E. (2018). Large-Scale Production of Stem Cells Utilizing Microcarriers: A Biomaterials Engineering Perspective from Academic Research to Commercialized Products. Biomaterials.

[136] Van Wezel A.L. (1967). Growth of Cell-strains and Primary Cells on Micro-carriers in Homogeneous Culture. Nature.

[137] Chen X.Y., Chen J.Y., Tong X.M., Mei J.G., Chen Y.F., Mou X.Z. (2020). Recent Advances in the Use of Microcarriers for Cell Cultures and Their Ex Vivo and in Vivo Applications. Biotechnol. Lett..

[138] Amer M.H., Alvarez-Paino M., McLaren J., Pappalardo F., Trujillo S., Wong J.Q., Shrestha S., Abdelrazig S., Stevens L.A., Lee J.B. (2021). Designing Topographically Textured Microparticles for Induction and Modulation of Osteogenesis in Mesenchymal Stem Cell Engineering. Biomaterials.

[139] Maciel M.M., Correia T.R., Henriques M., Mano J.F. (2022). Microparticles Orchestrating Cell Fate in Bottom-up Approaches. Curr. Opin. Biotechnol..

[140] Zheng S., Liu Q., He J., Wang X., Ye K., Wang X., Yan C., Liu P., Ding J. (2022). Critical Adhesion Areas of Cells on Micro-Nanopatterns. Nano Res..

[141] Darge H.F., Chuang S.H., Lai J.Y., Lin S.Y., Tsai H.C. (2021). Preparation of Thermosensitive PNIPAm-Based Copolymer Coated Cytodex 3 Microcarriers for Efficient Nonenzymatic Cell Harvesting during 3D Culturing. Biotechnol. Bioeng..

[142] Narumi Y., Iwai R., Takagi M. (2020). Recovery of Human Mesenchymal Stem Cells Grown on Novel Microcarrier Coated with Thermoresponsive Polymer. J. Artif. Organs.

[143] Li C., Qian Y., Zhao S., Yin Y., Li J. (2016). Alginate/PEG Based Microcarriers with Cleavable Crosslinkage for Expansion and Non-Invasive Harvest of Human Umbilical Cord Blood Mesenchymal Stem Cells. Mater. Sci. Eng. C.

[144] Zhou A., Ye Z., Zhou Y., Tan W.S. (2019). Bioactive Poly(ε-Caprolactone) Microspheres with Tunable Open Pores as Microcarriers for Tissue Regeneration. J. Biomater. Appl..

[145] Perteghella S., Martella E., de Girolamo L., Orfei C.P., Pierini M., Fumagalli V., Pintacuda D.V., Chlapanidas T., Viganò M., Faragò S. (2017). Fabrication of Innovative Silk/Alginate Microcarriers for Mesenchymal Stem Cell Delivery and Tissue Regeneration. Int. J. Mol. Sci..

[146] Shekaran A., Lam A., Sim E., Jialing L., Jian L., Wen J.T.P., Chan J.K.Y., Choolani M., Reuveny S., Birch W. (2016). Biodegradable ECM-Coated PCL Microcarriers Support Scalable Human Early MSC Expansion and in Vivo Bone Formation. Cytotherapy.

[147] Karimian S.A.M., Mashayekhan S., Baniasadi H. (2016). Fabrication of Porous Gelatin-Chitosan Microcarriers and Modeling of Process Parameters via the RSM Method. Int. J. Biol. Macromol..

[148] Huang L., Xiao L., Jung Poudel A., Li J., Zhou P., Gauthier M., Liu H., Wu Z., Yang G. (2018). Porous Chitosan Microspheres as Microcarriers for 3D Cell Culture. Carbohydr. Polym..

[149] Dosta P., Ferber S., Zhang Y., Wang K., Ros A., Uth N., Levinson Y., Abraham E., Artzi N. (2020). Scale-up Manufacturing of Gelatin-Based Microcarriers for Cell Therapy. J. Biomed. Mater. Res.-Part B Appl. Biomater..

[150] Smith D., Herman C., Razdan S., Abedin M.R., van Stoecker W., Barua S. (2019). Microparticles for Suspension Culture of Mammalian Cells. ACS Appl. Bio Mater..

[151] Xia P., Zhang K., Fang J., Yan S., Cui L., Chen X., Yin J. (2017). A Novel Fabrication of Open Porous Poly-(γ-Benzyl-L-Glutamate) Microcarriers with Large Pore Size to Promote Cellular Infiltration and Proliferation. Mater. Lett..

[152] Somville E., Kumar A.A., Guicheux J., Halgand B., Demoustier-Champagne S., des Rieux A., Jonas A.M., Glinel K. (2020). Green and Tunable Animal Protein-Free Microcarriers for Cell Expansion. ACS Appl. Mater. Interfaces.

[153] Roux R., Ladavière C., Montembault A., Delair T. (2013). Particle Assemblies: Toward New Tools for Regenerative Medicine. Mater. Sci. Eng. C.

[154] Tan Y.J., Tan X., Yeong W.Y., Tor S.B. (2016). Hybrid Microscaffold-Based 3D Bioprinting of Multi-Cellular Constructs with High Compressive Strength: A New Biofabrication Strategy. Sci. Rep..

[155] Liu S., Huang D., Hu Y., Zhang J., Chen B., Zhang H., Dong X., Tong R., Li Y., Zhou W. (2020). Sodium Alginate/Collagen Composite Multiscale Porous Scaffolds Containing Poly(ε-Caprolactone) Microspheres Fabricated Based on Additive Manufacturing Technology. RSC Adv..

[156] Qu H., Fu H., Han Z., Sun Y. (2019). Biomaterials for Bone Tissue Engineering Scaffolds: A Review. RSC Adv..

[157] Grebenik E.A., Grinchenko V.D., Churbanov S.N., Minaev N.V., Shavkuta B.S., Melnikov P.A., Butnaru D.V., Rochev Y.A., Bagratashvili V.N., Timashev P.S. (2018). Osteoinducing Scaffolds with Multi-Layered Biointerface. Biomed. Mater..

[158] Qu H. (2020). Additive Manufacturing for Bone Tissue Engineering Scaffolds. Mater. Today Commun..

[159] Demina T.S., Popyrina T.N., Minaeva E.D., Dulyasova A.A., Minaeva S.A., Tilkin R., Yusupov V.I., Grandfils C., Akopova T.A., Minaev N.V. (2022). Polylactide Microparticles Stabilized by Chitosan Graft-Copolymer as Building Blocks for Scaffold Fabrication via Surface-Selective Laser Sintering. J. Mater. Res..

[160] Zhou W.Y., Lee S.H., Wang M., Cheung W.L., Ip W.Y. (2008). Selective Laser Sintering of Porous Tissue Engineering Scaffolds from Poly(l-Lactide)/Carbonated Hydroxyapatite Nanocomposite Microspheres. J. Mater. Sci. Mater. Med..

[161] Liao H.-T., Lee M.-Y., Tsai W.-W., Wang H.-C., Lu W.-C. (2016). Osteogenesis of Adipose-Derived Stem Cells on Polycaprolactone- β -Tricalcium Phosphate Scaffold Fabricated via Selective Laser Sintering and Surface Coating with Collagen Type I. J. Tissue Eng. Regen. Med..

[162] Lin K., Liu J., Wu J.-M., Sun Y., Li F., Zhou Y., Shi Y. (2020). Selective Laser Sintered Nano-HA/PDLLA Composite Microspheres for Bone Scaffolds Applications. Rapid Prototyp. J..

[163] Chen C.-H., Lee M.-Y., Shyu V.B.-H., Chen Y.-C., Chen C.-T., Chen J.-P. (2014). Surface Modification of Polycaprolactone Scaffolds Fabricated via Selective Laser Sintering for Cartilage Tissue Engineering. Mater. Sci. Eng. C.

