# Correction: Sherstneva et al. Biodegradable Microparticles for Regenerative Medicine: A State of the Art and Trends to Clinical Application. *Polymers* 2022, *14*, 1314

**DOI:** 10.3390/polym17050622

**Published:** 2025-02-26

**Authors:** Anastasia A. Sherstneva, Tatiana S. Demina, Ana P. F. Monteiro, Tatiana A. Akopova, Christian Grandfils, Ange B. Ilangala

**Affiliations:** 1 Enikolopov Institute of Synthetic Polymeric Materials, Russian Academy of Sciences, 70 Profsouznaya Str., 117393 Moscow, Russia; sherstneva2912@mail.ru (A.A.S.); akopova@ispm.ru (T.A.A.); 2 Institute for Regenerative Medicine, Sechenov First Moscow State Medical University, 8-2 Trubetskaya Str., 119991 Moscow, Russia; 3 Interfaculty Research Centre on Biomaterials (CEIB), Chemistry Institute, University of Liège, B6C, 11 Allée du 6 Août, B-4000 Liege, Belgium; a.monteiro@uliege.be (A.P.F.M.); c.grandfils@uliege.be (C.G.); ange.ilangalabooka@uliege.be (A.B.I.)

## Insert New Acknowledgments

Acknowledgments were not included in the original publication [1]. The Acknowledgments section appears here.

**Acknowledgments:** The authors are grateful to Coralie Rocca for contributing to fruitful discussions.

The authors state that the scientific conclusions are unaffected. This correction was approved by the Academic Editor. The original publication has also been updated.
